# Action Augmentation of Tactile Perception for Soft-Body Palpation

**DOI:** 10.1089/soro.2020.0129

**Published:** 2022-04-19

**Authors:** Luca Scimeca, Josie Hughes, Perla Maiolino, Liang He, Thrishantha Nanayakkara, Fumiya Iida

**Affiliations:** ^1^ Bio-Inspired Robotics Laboratory, Department of Engineering, University of Cambridge, Cambridge, United Kingdom.; ^2^ Oxford Robotic Institute, University of Oxford, Oxford, United Kingdom.; ^3^ Morphological Computation and Learning Lab, Dyson School of Design Engineering, Imperial College London, London, United Kingdom.

**Keywords:** soft tissue palpation, soft haptics, soft tactile perception

## Abstract

Medical palpation is a diagnostic technique in which physicians use the sense of touch to manipulate the soft human tissue. This can be done to enable the diagnosis of possibly life-threatening conditions, such as cancer. Palpation is still poorly understood because of the complex interaction dynamics between the practitioners' hands and the soft human body. To understand this complex of soft body interactions, we explore robotic palpation for the purpose of diagnosing the presence of abnormal inclusions, or tumors. Using a Bayesian framework for training and classification, we show that the exploration of soft bodies requires complex, multi-axis, palpation trajectories. We also find that this probabilistic approach is capable of rapidly searching the large action space of the robot. This work progresses “robotic” palpation, and it provides frameworks for understanding and exploiting soft body interactions.

## Introduction

The palpation of soft bodies is a complex medical procedure where physicians palpate the human body for the diagnosis of abnormalities.^1,2^ Practitioners use their hands to explore and feel for abnormalities within the soft tissue of the patient's body, exploiting the physical structure and the sensing capabilities of the human hand.^3^ This action is widely used for the initial detection and screening of abnormalities within the body, aiding the diagnosis of conditions, including cancer,^4^ abdominal aortic aneurysm,^5^ appendicitis,^6^ and others.^7–9^

The complexity of this important procedure arises from the complex motions of the practitioner's hand that are in contact with the interacting layers of soft tissues of the human body, which can have many (or infinite) degrees of freedom. In the past, there have been notable attempts to better understand palpation by using robotics technologies.^10–13^ One of the pioneering works in this area, the WAPRO-4 system, is capable of performing simple breast palpation to identify relatively large inclusions.^14^ The use of probes with variable mechanical impedance has been found to improve lumps and tumor detection,^15^ and the importance of sensory-motor coordination has also been shown across a number of medical applications.^16–18^ One of the key enabling technologies to improve robotic palpation capabilities is tactile sensing,^19^ which has led to the in-depth study of the use of tactile sensors for tumor localization.^20–26^ Robotics research has also attempted the development of technologies for medical teleoperation^27–30^ and medical training, such as haptic palpation training systems,^31–34^ and virtual reality training systems.^7,35,36^ Finally, efforts have been made in applying machine learning for tumor localization and classification.^37–41^

Within this body of existing work, there has been limited investigation of the impact of introducing diversity and complexity into the trajectory of the robot hand/probe during palpation. Previous work has only examined robotics palpation systems with simple one-axis vertical displacements,^24,25,42,43^ or horizontal sliding trajectories.^25,34,44^ In contrast, medical practitioners use complex examinations techniques, including rotations, twists, and percussions that are dependent on the specific body part under investigation.^8^

In the context of palpation, the quality of the tactile information, and hence the ability to make accuracy diagnosis, depends on the quality of these soft interaction as well as the tactile information arising from them. In this article, we hypothesize that the tactile information gained through the interactions between a sensor and the soft human body is improved by introducing complexity into the robot actions. The robot actions can enhance the richness of the physical stimuli arising from the soft interactions between the robot and the soft body to palpate, assisting classification of inclusions and hence diagnosis. As such, the challenge addressed in this article is the optimization of complex palpation trajectories to enable more accurate classification of abnormalities in soft bodies.

We use a six Degree of Freedom robot arm with a sensorized end-effector. To efficiently search the high dimensional action space, we utilize Bayesian inference (in the form of Bayesian Exploration). Bayesian approaches can leverage the cumulative past experiences to rapidly search motion trajectory parameters, and they allow for efficient search of high-dimensional action spaces. This search can enable the robot to select effective trajectories for accurate classification of hard inclusions in soft tissues.

In this article, the Materials and Methods section briefly reports the physical set-up for the experiments, before outlining the Bayesian framework developed for this work. In the Materials and Methods section, we report the results. The Exploring Action Complexity in Robot Medical Palpation section shows the complex relationship between robot palpation trajectory and the ability to perform accurate diagnosis. Bayesian Approaches for Confident Abnormality The Detection section focuses on the use of our Bayesian framework to perform confident diagnosis; however, we show the ability of our framework to find optimal palpation strategies efficiently. The discussion and conclusion are finally reported in the Discussion and Conclusion section.

## Materials and Methods

**FIG. 1.**
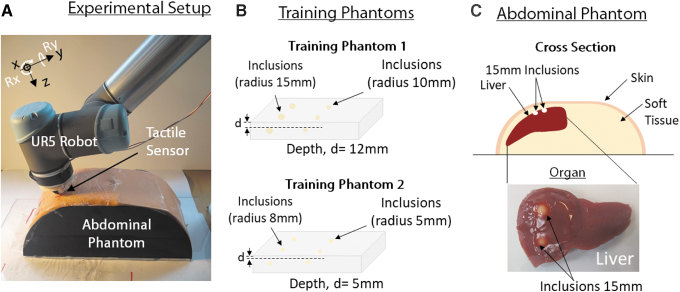
Robotic medical palpation, including **(a)** the experimental setup, **(b)** the training phantoms, and **(c)** the abdominal phantom developed.

The palpation experiments are performed by using a Robotic Arm with a sensing probe equipped with a capacitive tactile sensor array (Fig. 1). Although alternative sensor technologies could be used, the sensory technology chosen has a number of key advantages for use in palpation. The sensor provides pressure information from seven distributed “taxel” locations on the sensor surface, providing key spatial information with a high sensitivity, which is in line with that required for palpation.^45^ The taxels respond with a bell-shaped curve and their receptive fields overlap,^46^ allowing the detection of abnormal inclusions that are as small as 5 mm in diameter.

As this work focuses on the classification of hard inclusions, as opposed to their localization, we focus on point-based palpation trajectories, which revolve around a predefined point. This is in contrast to existing work, where the localization of inclusions was performed by using sliding trajectories.^25,34,44^ Each palpation experiment lasts 3 s, and, relative to the end-effector's initial position, the trajectory varies in depth axis (*Z*), rotation around the *x*-axis (*Rx*), and rotation around the *y*-axis (*Ry*). As such, each 3D trajectory can be described by six constant motion parameters (*A_rx_*, *A_ry_*, *A_z_*, *ω_rx_*, *ω_ry_*_,_ and *ω_z_*) (Supplementary Movie S1 and Supplementary Fig. S3).

The experiments are performed on three phantoms: two flat training phantoms, and a more human-like abdominal phantom (Fig. 1). Inspired by medical palpation of the liver, the Abdominal Phantom is a silicone phantom of a human liver embedded in a cross-sectional replica of a human torso. The replica introduces higher levels of complexity than the flat training phantoms, including a curved surface, skin, and tissues. All phantoms include stiff spherical inclusions of diameter 5, 10, or 15 mm, at a depth of 5 or 12 mm from the surface, as summarized in Figure 1b and c and Table 2. These sizes and depths mirror conditions in which inclusions are typically detected through palpation. More information about phantoms development can be found in the Phantom Development section in Supplementary Data (Supplementary Fig. S1).

**FIG. 2.**
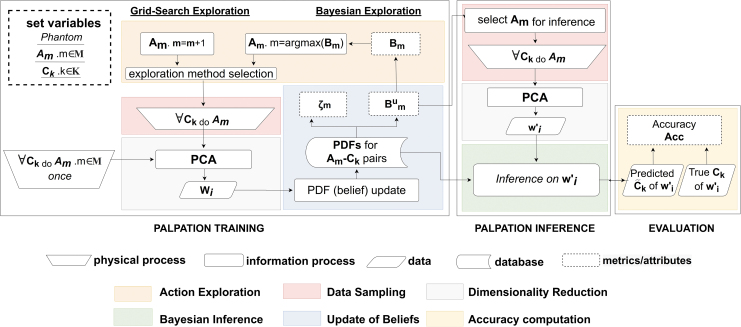
Flowchart of experimental procedure. During the palpation training phase, the robot performs palpations *A_m_* on different types of inclusions *C_k_* to form PDFs. After an initial set of palpations to generate PDFs, the robot performs additional experiments to improve its classification capabilities based on the biased *B_m_* score. In the palpation inference phase, the PDFs are used to perform inference on new samples. Moreover, an unbiased benefit and a confidence level for each palpation trajectory *A_m_* can be estimated. In the evaluation phase, the performance of the robot can be evaluated if the ground truth classification of the palpated area is known. PDF, probability density function.

Using this setup, we validate the need for complex motion strategies, and we find those strategies that can improve soft tactile perception for the classification of hard inclusions. The experimental framework we propose to achieve this has three key phases: training, inference, and evaluation (Fig. 2).

### Training phase

During the training phase, the robot generates sensory data by repeatedly palpating the different inclusions with different palpation trajectories. Each robot experiment involves a palpation trajectory, or action (*A_m_*), being performed on a specific class of inclusion (*C_k_*) in a phantom. These data are then represented probabilistically as probability density functions (PDFs).

#### Data sampling

**FIG. 3.**
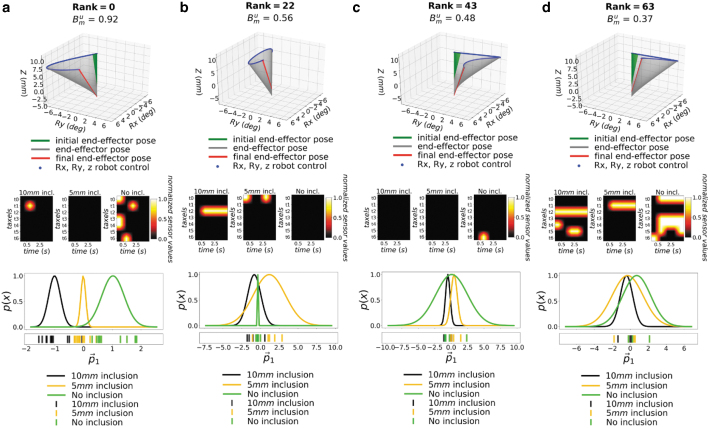
Influence of the palpation trajectory to the PDFs in *Training Phantom* 2. The 64 robot actions are ranked by Bum, and Rank 0-22-43-63 (best to worst) are shown, respectively, in **(a–d)**. The *top* plots show the robot trajectory generated during palpation, where *Rx*, *Ry* are rotations about the *x*-*y* axis and *Z* is the probing depth. The *middle* plots show the normalized spatiotemporal tactile images generated during palpation. The brightness corresponds to the normalized taxel values at specific time intervals (proportional to pressure). The *bottom* plots show the PDFs generated from 60% of the data detailed in the “Exploration Experiments” of Table 2. Here, *p*_1_ is the first principal component onto which the original sensor data were projected.

Let **X** be an *N* × *D* dimensional vector, where each unique temporal tactile image for a probed location is a *D*-dimensional row in the matrix. A temporal tactile image is a sequence of tactile images sampled at constant time intervals. Each tactile image corresponds to the normalized raw capacitance values of each taxel in the tactile sensor. By limiting the palpation to three seconds, we gain 35 pressure points over time limiting the dimensionality of the data (*D* = 35). The value of *N* is not constant, but it instead increases with the number of palpation experiments performed. In each experiment, the value of *N* is initially 0 and for each “palpation iteration” *N* = *N* + *K* where *K* is the number of discriminative classes, or types of inclusions in the phantom to palpate (examples of rows of **X** can be visualized as heatmaps in Fig. 3a–d). The final total number of experiments can be computed by the product of three variables, that is, (number of actions) × (number of inclusion classes) × (number of samples). Each of these three variables changes depending on the experimental conditions, and it is reported in Table 1. We use principal component analysis (PCA) to project the original tactile data **X** onto its first principal component *p*_1_, obtaining a matrix **W**, where each row *w_i_* is a one-dimensional (1D) projection of the original 35-dimensional tactile sensor data. A detailed explanation of the dimensionality reduction processes is provided in the Supplementary Data.

**Table 1.** Experimental Breakdown of Robotic Palpations and Palpated Phantoms

Phantom	Class of inclusions (mm)	Depth of inclusions (mm)	No. of inclusions	Complexity demonstration	Exploration experiments	Validation experiments	Total number
*Training Phantom 1*	10	12	3	1920 (10 iterations per trajectory)	3840 (20 iterations per trajectory)	—	5760
	15	12	3				
	No incl.	N/A	4				
*Training Phantom* 2	5	5	3	—	3840 (20 iterations per trajectory)	—	3840
	8	5	3				
	Healthy	N/A	4				
Abdominal test phantom	15 No incl.	12 N/A	2 2	—	800 (20 iterations per trajectory)		800

The values in parenthesis represent the number of samples gathered by the robot for any trajectory (*A_m_*) and class of inclusion (*C_k_*) pair.

N/A, not applicable.

**Table 2.** Experimental Breakdown of Robotic Palpation Trajectories and Parameters Over Experiments

	Complexity demonstration	Exploration experiments	Validation experiments
No. of trajectories attempted	64	64	20
Parameter combinations	*A_rx_* ∈ [0, $\frac{\pi }{18}$] *A_ry_* ∈ [0, −$\frac{\pi }{18}$] *A_z_* ∈ [0, 0.01] *ω_rx_* ∈ [0, 1] *ω_ry_* ∈ [0, 1] *ω_z_* ∈ [0, 0.5]	*A_rx_* ∈ [−$\frac{\pi }{18}$, $\frac{\pi }{18}$] *A_ry_* ∈ [−$\frac{\pi }{18}$, $\frac{\pi }{18}$] *A_z_* ∈ [0.002, 0.01] *ω_rx_* ∈ [1, 3] *ω_ry_* ∈ [1, 3] *ω_z_* ∈ [0.5, 2]	20 Highest score trajectories from “exploration experiments”

#### Bayesian PDFs update

Using the sensor data, Bayesian approaches can be applied, including Bayesian Exploration, an approach first proposed for tactile discrimination of textures,^47^ and other tactile discrimination tasks.^48,49^ In this article, we additionally derive a measure of confidence for each robot palpation trajectory. The mathematical details behind the representation proposed in this section can be found in Fishel and Loeb,^47^ as well as in the Bayesian Treatment of Sensor Evidence section.

To represent the sensor data probabilistically, we use the 1D tactile evidence computed through PCA to generate a probability density function (PDF) for each class of inclusion *C_k_*, and palpation trajectory *A_m_* via:

(1) $$p\left(w_{i}|C_{k},A_{m}\right)=\frac{1}{\sqrt{{\left(2\pi \right)}^{2}\left|\Sigma_{k,m}\right|}}e^{-\frac{1}{2}{\left(w_{i}-\mu_{k,m}\right)}^{T}\sum_{k,m}^{-1}\left(w_{i}-\mu_{k,m}\right)}$$

where (*μ_k,m_*) and (Σ_*k,m*_) denote the mean and standard deviation of the 1D sensor data from a series of palpation iterations. By representing the tactile sensor data probabilistically, the width of the PDF captures the variation of the sensor data for a given palpation action and inclusion type (Supplementary Movie S2).

From these training data, we can generate two key metrics that help assess the quality of a palpation trajectory. The first is an “Unbiased Benefit Estimator” **B**^*u*^_*m*_, which provides a measure of how useful the sensor data from a are for classification, and can be quantitatively measured by considering the overlap between the PDFs for the different classes of inclusions. This is based on the Bhattacharyya coefficient,^50^ which for two probability density functions *p* and *q* is defined as:

(2) $$BCoeff=\int \sqrt{p\left(x\right)q\left(x\right)}dx$$

Based on this coefficient, we can define the unbiased benefit estimator (**B**^*u*^_*m*_), for a specific action *A_m_* as:

(3) $$B_{m}^{u}=\sum_{k}^{K}\frac{P{\left(C_{k}\right)}^{2}}{\sum_{s}^{K}\Psi_{ks,m}P\left(C_{s}\right)}$$

where Ψ_*ks,m*_ is calculated from the Bhattacharyya coefficient, and contains the mutual confusion between two classes and *C_k_* and *C_s_* are under action *A_m_*. *P*(*C_k_*) and *P*(*C_s_*) are the prior probabilities of inclusions *C_k_* and *C_s_*, respectively. This **B**^*u*^_*m*_ score can be used to rank palpation trajectories. Using the properties of Gaussian distributions, a lower degree of overlap among distributions implies a higher likelihood for any new sample to fall into an area of sensor space belonging to either class unambiguously. As such, a higher overlap should imply a higher prediction accuracy.

One of the unique advantages of computing an unbiased benefit estimator is that we can also obtain a measure of confidence of the tactile sensor data for a specific palpation trajectory *A_m_*. This measure of confidence, *ζ_m_*, is defined as:

(4) $$\zeta_{m}=1-\frac{1}{2+e^{-B_{m}^{u}}}$$

This metric increases monotonically when the discriminatory confusion reduces, and it signifies classification confidence for a specific trajectory.

#### Exploratory action identification

As the number of parameters that describe a palpation trajectory increases, there is an exponential increase in the number of actions to be searched. If each of the six action parameter can take on “*n*” possible values, there are up to *n*^6^ trajectories to search. Efficient search *i* approaches are required, as it is neither practical nor feasible to perform searches of this scale for each new phantom or patient. Figure 2 illustrates how Bayesian Exploration can be implemented to find optimal palpation actions by iteratively selecting, and exploring, the most “promising” action.^47^ This search requires a metric to guide the selection of actions. For this we can use a “biased” benefit score based on **B**^*u*^_*m*_, that is:

(5) $$B_{m}=1-{\left(1-B_{m}^{u}\right)}^{\frac{1}{n_{m}}}$$

where *n_m_* is the number of times action *m* was performed iteratively during the palpation experiments. Thus, the biased benefits are discounted by the number of times the action has already been performed during action exploration to discourage excessive exploitation and eventually encourage the explorative update of belief states under less exploited actions.

Initially, the robot palpates each class of inclusion under every action once, to gather initial experimental evidence. After this, the action is selected by using **B**_*m*_, each class is palpated by using this action, and the PDFs are then updated accordingly.

### Bayesian inference phase

In the second phase of the framework, Bayesian inference, the robot performs the classification of abnormal inclusions, identifying the class of unseen sensor data obtained through additional robotic palpations. This classification is made via Bayesian Inference, using the PDFs generated in the palpation training phase through several palpation iterations. To perform inference on a new tactile sample *w_i_*^0^, we evaluate the sample at $p\left(w\prime_{i}|C_{k},A_{m}\right)$, under every *C_k_* for a chosen action *A_m_*. The *C_k_* of the PDF yielding the highest value will be inferred as a class for *w_i_*^0^.

(6) $${\tilde{C}}_{k}=argmax\left\{p\left(w\prime_{i}|C_{k},A_{m}\right):k\in C\right\}$$

where *C*_e*k*_ is the class estimated for *C_k_*. This inference process is used throughout the results section to test the abilities of different palpation trajectories, and it will be referred to as “Bayesian inference classification.”

### Evaluation phase

In this final phase, we evaluate the performance of the classification by comparing the “true” class of inclusion *C_k_* against the class that was inferred. Over several iterations, we can count the number of correctly classified abnormal inclusions as True Positives, and the number of correctly classified inclusion-free areas of the phantom as True Negatives. For a total of *N_C_* classifications, or palpation inferences, the accuracy can be formally computed as:

(7) $$Acc=\frac{TP+TN}{N_{C}}$$

## Results

### Exploring action complexity in robot medical palpation

The first set of experiments investigates the influence of the palpation trajectory on soft tactile sensing capabilities and the ability to distinguish different classes of inclusion.

In these experiments, we examine 64 different palpation trajectories, and we analyze how they influence the separation of PDFs. The 64 palpation trajectories are generated through the combination of six parameters that describe the trajectory (2^6^ = 64). We conducted these experiments on *Training Phantom 1*, performing all the palpation actions on all the different inclusion types. For each type of inclusion and palpation trajectory, we perform the palpation 20 times. This brings the total number of experiments to 64 × 20 × 3, where 3 is the number of classes of inclusions present in the phantom. More details can be found in the “Exploration Experiments” column of Tables 1 and 2.

Figure 3 shows the PDFs for four exemplar palpation trajectories, ordered with respect to the **B**^*u*^_*m*_ scores. The PDFs are created via Equation (1), and the unbiased benefit score **B**^*u*^_*m*_ is computed for each palpation action by using Equation (3). The different motion parameters result in different palpation trajectories with very diverse PDFs. In Figure 3, an example of reference raw tactile sensor data of the inclusions is shown in the middle figures, where the *y*-axis represents a layout of all taxels (Supplementary Fig. S2), and the *x*-axis the experiment time. The corresponding PCA projected points are shown in the *x*-plot of the lower figures, together with their corresponding PDFs. The raw tactile sensor data for each class are influenced by the palpation strategy itself. Ideally, PDFs for different inclusion classes should have minimal overlap for discrimination purposes. The results show how it is possible to have motion parameters, and hence trajectories, that give rise to PDFs that are fully separated across the PCA principal component *p*_1_ for all classes of inclusion (Fig. 3a).

The figure also shows that the degree of these overlaps can be represented by using the **B**^*u*^_*m*_ scores. The trajectories with less overlap (Fig. 3a, b) result in higher **B**^*u*^_*m*_ scores, whereas those with more overlap (Fig. 3c, d) have a far lower score. As such, the score represents the discriminative performance of the palpation trajectory and can be used to compute a ranking for the different trajectories. **B**^*u*^_*m*_, also indicates the degrees to which each PDF is separated from the others, in addition to measuring the amount of overlap. This allows similarly overlapped PDFs to be ranked; for example, although the PDFs of the 43rd and 63rd ranked actions are similarly overlapped, the former is ranked higher because of the lower overlap between the PDFs of the 10 and 5 mm inclusion classes.

In the next experiment, we compare the separation of the PDFs for the same trajectories but across different phantoms, that is, *Phantom 1*, *Phantom 2*, and the *Abdominal Phantom*. We perform this experiment to assess whether a palpation trajectory optimized for one phantom can perform well on other phantoms.

**FIG. 4.**
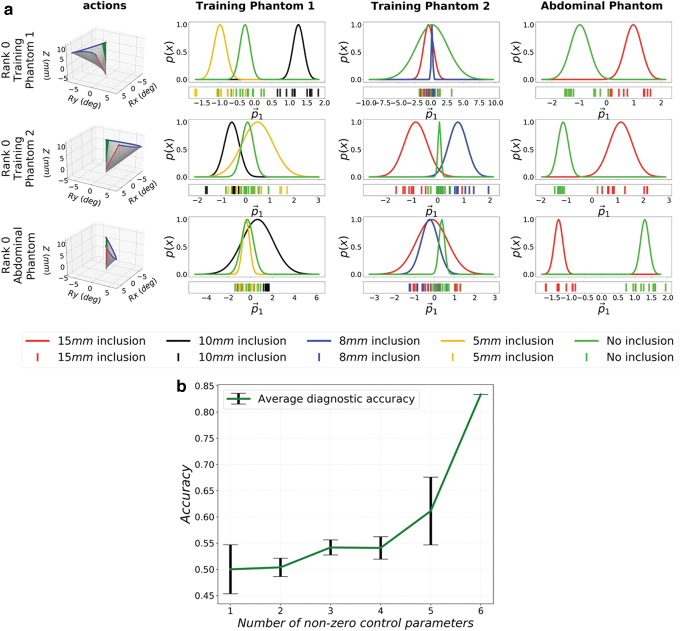
The figure shows the complexity of robotics palpation. The diagonal plots in **(a)** show the PDFs of the best performing palpation trajectories for each phantom, whereas the off-diagonal plots show PDFs of the same trajectories in all other phantoms. **(b)** Shows the accuracy of a Bayesian Inference classifier trained on sensor data generated via palpation trajectories with a varying number of parameters.

To achieve this, we identified the best trajectories for *Phantom 1*, *Phantom 2* and the *Abdominal Phantom*, by finding the trajectory with the highest **B**^*u*^_*m*_ score for each phantom. These three top-ranking trajectories, together with the resulting PDFs, are compared in Figure 4a. The experimental data used are detailed in the “Exploration Experiments” of Tables 1 and 2. The first observation is that the optimum trajectories are significantly different for the different phantoms. The best palpation trajectory for *Phantom 1* is a counter clockwise rotation in an almost horizontal plane, whereas that for *Phantom 2* is a clockwise trajectory with a similar amplitude. The optimum trajectory for *Abdominal Phantom* is significantly different, with a clockwise rotation occurring with smaller amplitude, and a higher palpation depth. The second observation that can be made considers the PDF overlaps. Figure 4a shows that the highest ranked actions do not show high separation of the PDFs on the other phantoms. The best trajectory for *Phantom 1*, for example, does not perform well in *Phantom 2*, with the action resulting in high overlaps of PDFs belonging to different classes of inclusions. The *Abdominal Phantom* is a relatively easier task, in comparison to *Phantom 1* and *Phantom* 2, with all of three palpation strategies achieving high separation of the PDFs. However, the trajectory ranking higher for *Abdominal Phantom* still achieves higher separation of PDFs in the same phantom, whereas it does not perform well in *Phantom 1* and *Phantom 2*.

In the next set of experiments, we examine the need for more complex trajectories for more accurate palpation of soft bodies. This is achieved by comparing palpation trajectories that are described by a different number of control parameters. As each axis of motion is controlled by a specific pair of parameters (i.e., *A_rx_*-*ω_rx_*, *A_ry_*-*ω_ry_*, and *A_z_ω_z_* to control *Rx*, *Ry*, and *Z*, respectively); reducing the number of parameters decreases the complexity of the trajectory.

To systematically vary and reduce the complexity, every possible combination of the six parameters is set to zero in turn. The 64 palpation trajectories defined by these parameters are performed 10 times on all types of inclusions in *Training Phantom 1*, and the corresponding tactile data are stored. These data correspond to tactile information from 2160 palpations; these are depicted in the columns of Tables 1 and 2.

To evaluate the performance of each set of motion parameters, Bayesian Inference classification is performed on the computed PDFs. The classification inference is performed on each palpation trajectory separately, with 60% of the sampled palpations used for training, and the remaining 40% used for testing. Figure 4b shows the average performance of the classifier across all palpation trajectories with different numbers of active parameters. As illustrated in Figure 4b, trajectories described by one or two parameters achieve accuracy rates of 50% on average, thus a little above random selection (33%). With the full employment of the six descriptive parameters, the generated trajectories can achieve accuracies above 60%. As shown in Figure 4b, when the dimensionality of the actions, and hence number of motion parameters, is increased, there is up to 35% improvement in the average classification accuracy of the robot. This justifies and demonstrates the need for complex trajectories when performing palpation.

From this first set of experiments, we can make several conclusions. First, the palpation trajectory influences the tactile sensor data significantly, where, slight changes in the palpation trajectory can significantly affect the discriminatory abilities of the robot. Second, the optimum trajectories vary from phantom to phantom. There is not one “optimum” motion for all phantoms. Third, introducing more complex palpation trajectories allows for better action profiles to emerge, demonstrating that increasingly complex actions increase the ability to make more accurate classification of abnormalities in soft bodies.

### Bayesian approaches for confident abnormality detection

The next set of experiments examines the levels of confidence (*ζ_m_*) and the experimental accuracy (*Acc*) when computing the PDFs based on a different number of training samples.

In these experiments, the same dataset from the previous experiments was used, where palpation training was performed on each class of inclusion, using each action 20 times (see “Exploration Experiment” columns of Tables 1 and 2).

Out of the 20 palpation samples for each class-action pair, 40% of the data (corresponds to 8 samples) is held out for testing. For every trajectory, then, we consider the remaining 12 samples and compute the PDFs with a varying number of samples, from 1 to 12. Every time the PDFs are computed, we also compute the benefit and the confidence as previously described. The resulting PDFs are also used to compute the accuracy, as described in Materials and Methods Section.

**FIG. 5.**
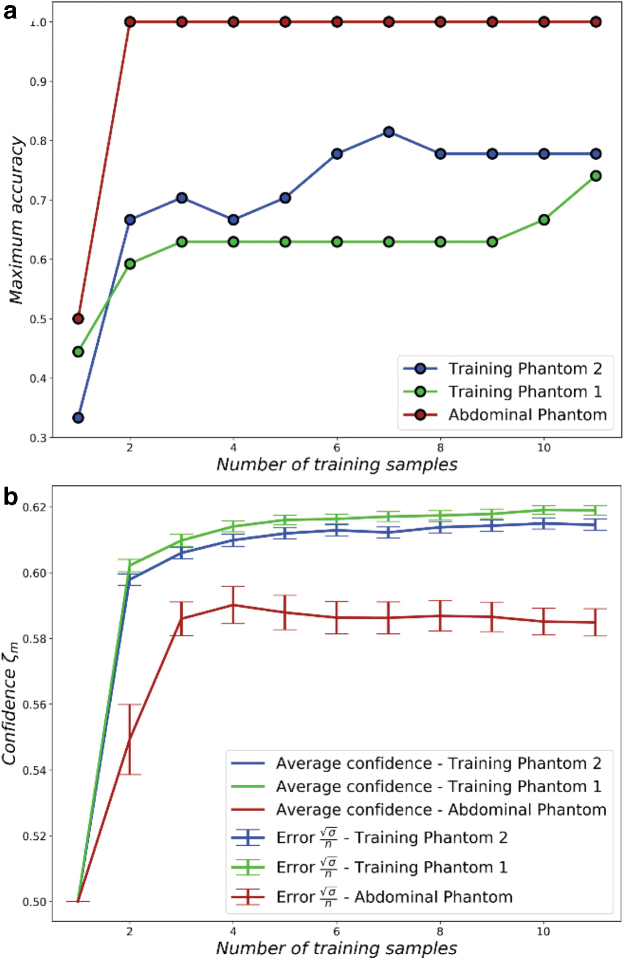
The figure shows the performance of a Bayesian inference classifier within the framework developed. **(a)** Shows the relationship between the maximum classifier accuracy and the number of samples gathered for each palpation trajectory-class pair. **(b)** Shows the relationship between the developed confidence level and the number of samples gathered for each palpation trajectory-class pair. The *vertical bars* in the plot illustrate the errors of the confidence at that point.

We show that it is possible to achieve high classification accuracy if appropriate actions are selected. Figure 5a shows the highest accuracy of all palpation trajectories, as a function of the number of training samples used to compute the PDFs. As the number of training samples increases, the evidence used to build the PDFs increases, leading to the best classifiers performing more accurate classification. In this set of experiments, the robot is also observed to reach maximum classification accuracy on the *Abdominal Phantom* by employing actions with more than two parameters.

In Figure 5b, the confidence metric is also plotted as a function of the number of samples used for training. As expected, we can see that the confidence metric increases with the number of training samples. The confidence, however, saturates at different values for each phantom. These values indicate how “reliable” the classification of the robot is under a specific trajectory. This measure will first and foremost depend on the overlap of the PDFs, which will, in turn ,depend on the similarity of the tactile sensor data for different classes of inclusions. In Figure 5b, the robot achieves highest confidence for *Phantom 1*, followed by *Phantom 2* and the inclusion-action pair. This initial gathering of evidence allows the Bayesian Exploration process to then start (Fig. 2).

In this exploration process, all palpation trajectories are ranked by using the biased *B_m_* score. The action that has the highest *B_m_* score is then used to palpate each class of inclusion once, and the PDFs are updated with the new tactile information. This corresponds to one iteration of the Bayesian Exploration framework. The *B_m_* score is then computed again and used to select the next palpation trajectory to test, with the steps then iteratively repeated. To evaluate each iteration of the exploration process, we take the top scoring action at that time, as defined by the unbiased benefit score, and use this action perform Bayesian Inference. The inference is performed on 40% of unseen data from “Exploration Experiments,” and it provides the robot with the “best accuracy” for every iteration of the exploration process. Importantly, the top scoring action is selected by the unbiased benefit score, as we want to find the top performing action that is purely based on the ability to separate the PDFs in sensor space. As a benchmark, the results from the grid-search method are also presented. During grid search, contrarily to Bayesian Exploration, the action is selected based on a breadth-first parametric search, with the rest of the experiments performed in the same manner.

**FIG. 6.**
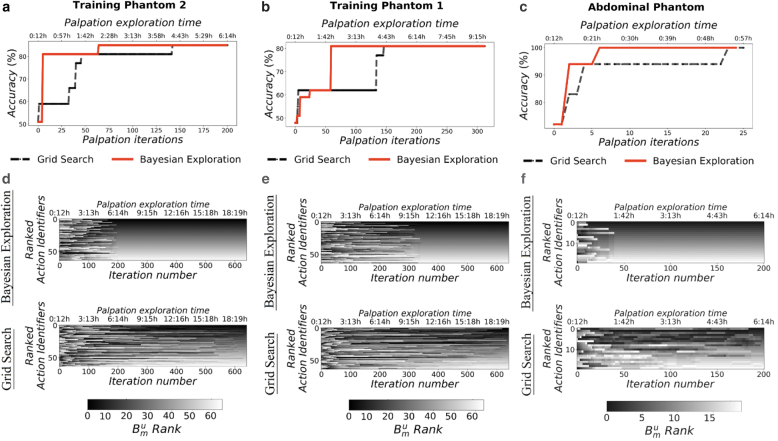
Training comparison between Bayesian online exploration and Grid Search. **(a–c)** Show the maximal accuracy achieved at each training iteration by both Grid Search and Bayesian Exploration, on a left-out validation set of eight palpation samples for every class of inclusion. **(d–f)** Show the trajectories on the *y*-axis, ordered based on their final **B**^*u*^_*m*_ rank during training for both Bayesian Exploration and Grid Search. The color intensity for each action indicates its rank at each iteration. Bayesian Exploration achieves a final ranking faster, bringing the robot's trajectories to a ranked solution in at least half the time than when training through a systematic action space search.

We compare the performance of these methods by considering the number of “palpation iterations” necessary to train the robot. As previously described, a “palpation iteration” involves the palpation of all classes of inclusions *C_k_* under a specific action *A_m_*. The action *A_m_* is here iteratively selected through Bayesian Exploration or Grid-Search. As shown in Figure 6a–c, Bayesian Exploration achieved its highest performance after around only 60 iterations in both training phantoms. On the *Abdominal Phantom* this took ∼7 iterations. Conversely, a grid-based systematic search performed poorly, finding equally good palpation strategies after 150 palpation iterations on the training phantoms, and 23 iterations on the *Abdominal Phantom.*

In Figure 6d–f, the intensity of the color shows the final ranking of the actions. The figure shows how this ranking is “unstable” for grid search, that is, the ranking keeps changing throughout the experiments, before reaching the final rank. Bayesian Exploration, however, induces a stable ranking much sooner, where the final ranking of trajectories is found much earlier on in the experiments.

By applying Bayesian Exploration, and leveraging the ranking provided by the score, the actions that best separate the PDFs across different classes of inclusions are preferentially explored. By using this exploration technique, the robot can efficiently search a high-dimensional parameter space. This complex high-dimensional action space has previously been demonstrated to be necessary for accurate classification of abnormal inclusions in soft tissues. From these results, we can observe that by using Bayesian Exploration, the time taken to find the optimal strategy is halved in comparison to a systematic grid search.

Finally, after performing Bayesian Exploration, we can report the final accuracy of the entire framework across all palpated phantoms. As previously explained, this is computed as the accuracy achieved on 40% of unseen palpation samples from each phantom. Table 3 reports the final highest test accuracy observed after training. Since the hypothesis in this article hinges on the postulate that appropriate palpation trajectories can aid in abnormality detection via palpation, we also report the average accuracy across all attempted palpation trajectories in Table 3. These results show how on average the palpation trajectories perform quite poorly, and appropriate optimization procedures are necessary to find the highest performing palpations. This highlights the important of Bayesian Exploration in this context.

**Table 3.** Highest and Average Classification Accuracies Achieved by the Palpation System When Training the Bayesian Classifier on 14 Samples of Each Class of Inclusion and Testing on Six Unseen Samples

Accuracy % highest (average)	15 mm vs. NA	10 mm vs. NA	8 mm vs. NA	5 mm vs. NA	15 mm vs. 8 mm vs. NA	10 mm vs. 5 mm vs. NA
*Training Phantom* 1	0.944 (0.462)		0.778 (0.394)		0.740 (0.412)	
*Training Phantom* 2		0.889 (0.438)		0.833 (0.387)		0.778 (0.423)
*Abdominal phantom*	1.0 (0.85)					

NA, no inclusion.

Notably, the system is capable of achieving more than 80% accuracy when discriminating between 5 mm inclusions and no inclusions. On the *Abdominal Phantom*, the robot achieves 100% accuracy when discriminating between 15 mm inclusions and no inclusions. Moreover, the highest performing motion strategies outperform the average performance of any one action by approximately a factor of two in almost all scenarios, confirming and emphasizing the need for appropriate palpation trajectories during abnormality detection.

## Discussion and Conclusion

Medical palpation is an impactful preliminary diagnosis tool that is used widely by primary care physicians, yet it is extremely challenging for a robot to perform due to the complexity of the interactions. The interactions between the palpation device and the soft human body are nonlinear; the complexity of the action space and the interactions is significant; and the solutions are different for every “patient.” Thus, to gain a more insightful understanding of this problem, we need to go beyond typical robotic approaches, including modeling and optimization. In this work, we perform large-scale physical experiments to understand whether and how multi-axis palpation trajectories can influence a robot's soft tactile perception to make accurate classification of abnormal inclusions in soft bodies. The framework presented in this work (Fig. 2) allows for the fast exploration of a high-dimensional action space, which arises from the palpation of soft bodies. The framework identifies palpation strategies that allow for a confident classification of the presence, or absence, of abnormal inclusions. The identified palpation strategies have been shown to enable the confident detection of abnormal inclusions that are as small as 5 mm in diameter (Table 3).

In this experimental approach to palpation, we have identified that increasing the complexity of the palpation trajectory can be beneficial for soft tactile perception in the context of palpation. In addition, we have shown that slight changes in the trajectory, or the patient, significantly affect the performance. This demonstrates that the optimum palpation trajectory must be found or identified for each patient through physical experimentation, and mirrors the method in which human practitioners find the best palpation motion for each patient. To make intelligent decisions in this soft, nonlinear, and highly complex space, we have demonstrated how a probabilistic Bayesian approach allows for accurate and efficient search and decision making. However, the parameterization of the trajectory is still based on human design and intuition, and as such, they are limited. In future scenarios, the parameterization and trajectories would ideally emerge from the haptic interaction with the soft tissue itself.

Going forward, this knowledge is important in several ways. In the long term, we can use the methods to develop “robot doctors” who can perform accurate and confident diagnosis. The framework development provides a starting point for the experiment procedure for such a robot. However, to achieve this, it is necessary to find appropriate ways to perform knowledge transfer across patients or phantoms. In the short term, we can use this understanding to improve robot tactile sensing in soft environments/settings. We can also apply the methods and approaches to other similar problems, where the Bayesian treatment and large-scale physical experiments would further our understanding of the problem at hand.

Finally, it would be interesting to explore the relationship between the investigated, point-based, palpation trajectories and sliding trajectories explored for localization.

## Supplementary Material

Supplemental data

Supplemental data

Supplemental data

Supplemental data

Supplemental data

Supplemental data
