# Supplementary material for: Action Augmentation of Tactile Perception for Soft-Body Palpation
Source: Soft Robot. 2022 Apr 19;9(2):280–92. doi: 10.1089/soro.2020.0129 (PMC9347261; doi:10.1089/soro.2020.0129)
Supplement: Supplemental data [file Supp_FigS3.docx]

**Figure S3.** Execution and parameterization of the robot control, and palpation trajectory. a) Diagram of the end-effector robot control during the palpation, (b) Example robot control strategy over time, given the parameters: *A_rx_*, *A_ry_*, *A_z_*, *ω_rx_*, *ω_ry_* and *ω_z_*, (c) Corresponding generated robot trajectory over the 3s palpation.
