# Supplementary material for: Action Augmentation of Tactile Perception for Soft-Body Palpation
Source: Soft Robot. 2022 Apr 19;9(2):280–92. doi: 10.1089/soro.2020.0129 (PMC9347261; doi:10.1089/soro.2020.0129)
Supplement: Supplemental data [file Supp_Data.docx]

Action Augmentation of Tactile Perception for Soft-Body Palpation

Luca Scimeca and Josie Hughes and Perla Maiolino and Liang He and Thrishantha

Nanayakkara and Fumiya Iida ^^[[1]](#footnote-1)^^

# **SUPPLEMENTARY MATERIALS**

## A.1. Phantom Development

To provide a physical test bed for the abnormality detection experiments several phantoms were created. The phantoms were created by casting Eco-Flex 00-10 Silicone and included a number of spherical 3D printed beads of different sizes and depths. The stiffness of the silicone (6.7 kPa) was chosen to be close to that of human tissue.

The casting of the phantoms included an initial casting of silicone of ‘d’ height at the bottom of a custom cuboidal mould. The initial cast layer would become the top layer of the phantom. After curing, 3D printed inclusions of various sizes are then glued into place on the phantom using a minimal amount of superglue. The beads were placed away from the edges, and with sufficient area around to provide area of the phantom free of any abnormal inclusions (no-inclusion). To complete the process, a second layer of silicone was cast, this time approximately 30 *mm* in height. This depth was chosen so that the dynamics of the palpation experiments would not be dominated by the interaction between the phantom and the surface on which this was placed. After curing, the phantoms could then be released from the moulds. To minimize damage, and extend their usable life, a thin film of cling film was then applied to their surface.

Two training phantoms were developed to test different palpation difficulties in the identification of inclusions in human bodies, and kidneys in particular. The inclusion in Training *Phantom 1* varied from 8 *mm* to 15 *mm* in diameter size and were set at a depth of 10 *mm*, a typical size and depth to tumours which may be identified on the kidney. To demonstrate how the framework developed could also be used to identify smaller, more superficial tumours, a second phantom, *Phantom 2* was created which had inclusions of size 5 *mm* and 10 *mm*, and at a depth of 5 *mm*.

To demonstrate the adaptability of the framework in complex and realistic environments, an *Abdominal Phantom* was created. This was designed to include a silicone kidney organ with inclusions and structure replicating the surrounding tissues and skin. Fig. 1 demonstrates the process of creating the phantom. The approach to fabrication was adapted from previous work.^51^ The phantom was developed by first fabricating a liver from Eco-flex 00-10 and red dye using a 3D printed mould. Inclusions were added to this liver, and additional silicone used to seal these inclusions. A second mould was then used to cast the abdomen using Ecoflex 00-00, this was done in multiple layers to allow the liver to be positioned in the correct place and with the correct curvature. To create a skin Ecoflex 00-20 was cast into a flat sheet with approximately 1*.*5 *mm* thickness which was then used to cover the phantom.

## A.2. Sensor Technology

The sensor module has a layered structure consisting in: a Flexible Printed Circuit Board (FPCB) which hosts 7 tactile elements (Taxels), corresponding each to the first plate of a capacitor, and a Capacitance to Digital Converter (CDC AD747 from Analog Devices); a dielectric layer (in this case made by an air chamber); and conductive lycra which act as common ground plane for all the taxels and constitute the second plate of the capacitor.

The taxels have a diameter of 4 *mm* and a uniform spatial placement with a pitch of 7 *mm* (Fig. 2). The module is connected to a microcontroller board (Intelligent Hub board - IHB) which collects taxel measurement through an SPI bus and processes them before sending them to the PC through CAN-BUS. The sensor provides measurement with a resolution of 16 bits corresponding to a variation of capacitance proportional to the pressure acting on top of the sensor and is sampled at 50*Hz*. A sensor reading, or tactile image, from the tactile sensor corresponds to a 7-dimensional array, where each element contains the capacitance variation value of the corresponding taxel. Fig. 2 shows the full sensor architecture.

The sensor has previously been integrated into a number of existing robotic systems which exploit sensory-motor co-ordination^52,53^ and soft tissue palpation.^54^

## A.3. Robot Control

As the work focuses on the classification of hard inclusions, as opposed to their localization, we focus on point-based palpation trajectories. A palpation experiment thus consists of *N_s_* seconds of contact between the sensorised robot end-effector with a target phantom location. To achieve this, we manually teach the robot the location of the phantoms areas to palpate, and set the palpation starting position with the end-effector alighted normally to the surface of the phantom organ (Fig. 3a). The end-effector is thus driven downward until a touch event is detected by the capacitive tactile sensor at its extremity, whereby the palpation experiment begins.

The parameterization of the robot control action was designed to generate point-based palpation trajectories, which revolve around a single defined point, whilst maintaining freedom of wrist rotations and probing depth.

The end-effector of the robot was controlled in real-time in Cartesian coordinates, acting upon the depth (*z* tool axis), *Rx* (rotation around the x tool axis), and *Ry* (rotation around the y tool axis) axis simultaneously. Distinct sinusoidal displacements profiles are generated for every axis, each of which is controlled by two separate parameters, thus a total of 6 parameters were used to control the robot for each palpation procedure, i.e. *A_rx_*, *A_ay_*, *A_z_*, *ω_rx_*, *ω_ry_* and *ω_z_*. For an arbitrary axis ‘*ax*’, a sinusoidal displacement *s_ax_*(*t*) over the course of the palpation experiment is defined as:


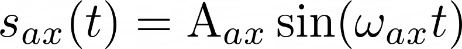
 (8)

where ‘*t*’ is the time, in seconds, elapsed since the start of the palpation experiment, and *A_ax_* and *ω_ax_* the axis-dependent parameters (Fig. 3 b,c). The achieve the displacement profiles, a UR5 robotic arm was speed controlled at 60Hz via:


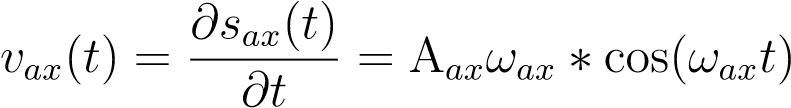
 (9)

where *v_ax_*(*t*) is the axis velocity in ‘*ax*’ at time ‘*t*’. Two values for each of the six parameters were explored, for a total of 64 different available palpation techniques. The *Z* parameters were set to drive the end-effector into the flesh of the silicone at a variable height between 2 *mm* and 10 *mm*, thus [A*_z_* ∈ [0*.*002*,*0*.*01]] and [*ω_z_* ∈ [0*.*5*,*2]]. The parameters for the *Rx* and *Ry* rotations were set to achieve variable rotations between ±10 degrees, thus [*ω_rx_,ω_ry_* ∈ [1*,*3]] and
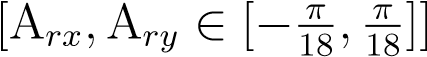
. The rotations and depths were chosen as the maximal achievable end-effector rotations and probing depths within all phantoms and inclusions under examination, without reaching the saturation of the tactile sensor response.

## A.4. Dimensionality Reduction

After obtaining the tactile image sequences matrix X, we use Principal Component Analysis to reduce the dimensionality of the high dimensional spatiotemporal palpation evidence.^55^ The average tactile sequence for each palpation can be computed as:


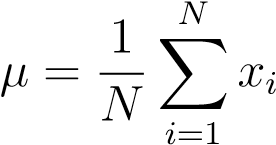
 (10)

where *x_i_* is a column vector corresponding to the i*^th^* row in X. We compute a (*D* × *D*) scatter matrix S as:


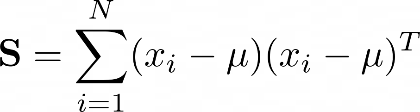
 (11)

and use Single Value Decomposition to factorize S into


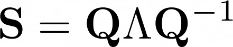
 (12)

where Q is a matrix such that each column *q_j_* corresponds to an eigenvector of *S*, and each element *λ_jj_* in the diagonal matrix *λ* is its corresponding eigenvalue. We list the eigenvectors in descending order of eigenvalue and select the first in the list. Let *p*_1_ be the selected eigenvector obtained from PCA. We form a (*D* × 1) projection matrix
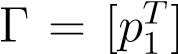
 where
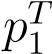
 is a column vector in Γ. Finally, we project the D-dimensional row vectors in X onto a 1-dimensional subspace by:


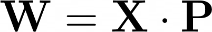
 (13)

where **W** is a (*N* ×1) matrix, and each row in the matrix is a 1-dimensional encoding of a tactile image sequence for a probed location. We will refer to as a generic projected tactile sensor measurement of a probed location after palpation.

*A.5. Bayesian Treatment of Sensor Evidence:*

The Bayesian treatment of the sensor evidence follows the Bayesian Exploration framework first proposed in ^47^ , with the additional implementation of a measure of confidence for each robot palpation trajectory. For each palpation experiment on a specific phantom, the class of inclusion under palpation (*C_k_*) and the type of palpation action (*A_m_*) generate an observable sensor measurement *w_i_*. The likelihood that a specific inclusion class *C_k_* ∈ *C* has generated the haptic observation *w_i_* can be computed as:


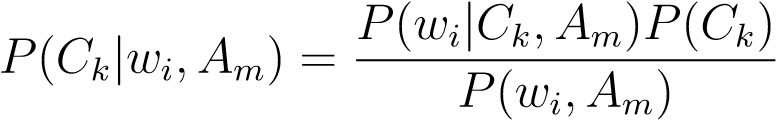
 (14)

where *A_m_* is a specific palpation action and *P*(*C_k_*) is the prior probability of inclusion *C_k_*.

The prior probabilities for each class can be extracted from the domain knowledge of the task to perform. For breast cancer detection, for example, the prior probability of being diagnosed with cancer can vary between 0.0017 and 0.0102 depending on risk factors,^56^ while for the experiments within this manuscript there is an equal probability of tissues areas with and without abnormal inclusions, thus for all classes. To find the probability of observing *w_i_* when performing a palpation *A_m_* on a class *C_k_*, instead, the central limit theorem suggests that we can approximate the findings with the probability density function *p*(*w_i_*|*A_m_*) defined by a mean *µ_k,m_* and a standard deviation Σ*_k,m_* as:


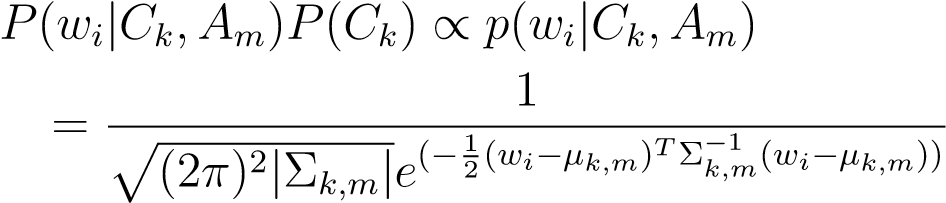
 (15)

The remaining steps to apply Bayesian Exploration are detailed in the main text.

**Resources:** All data and code can be found at: https://github.com/lucascimeca/robotics_palpation

1. Luca Scimeca, Josie Hughes and Fumiya Iida are with the Bio-Inspired Robotics Laboratory, Department of Engineering, University of Cambridge, Cambridge CB2 1PZ, U.K., Perla Maiolino is with the Oxford Robotic Institute, University of Oxford, Oxford OX2 6NN, U.K. and Liang He and Thrishantha Nanayakkara are with the Morphological Computation and Learning Lab, Dyson School of Design Engineering, Imperial College London, London SW7 1AL, U.K. [↑](#footnote-ref-1)
