# Supplementary material for: Action Augmentation of Tactile Perception for Soft-Body Palpation
Source: Soft Robot. 2022 Apr 19;9(2):280–92. doi: 10.1089/soro.2020.0129 (PMC9347261; doi:10.1089/soro.2020.0129)
Supplement: Supplemental data [file Supp_FigS1.docx]

# A. SUPPLEMENTARY MATERIALS

**Figure S1.** Process for creating the *Abdominal Phantom*, showing the curing of abdomen and adding the inclusions and the overall construction of the abdomen.
