# Supplementary material for: Action Augmentation of Tactile Perception for Soft-Body Palpation
Source: Soft Robot. 2022 Apr 19;9(2):280–92. doi: 10.1089/soro.2020.0129 (PMC9347261; doi:10.1089/soro.2020.0129)
Supplement: Supplemental data [file Supp_FigS2.docx]

**Figure S2.** Sensor Technology and Robot Control. (A) CySkin sensor patch used for the experiments (B) CySkin architecture within the robotics set-up for the experiments.
